# Partial replacement of cement with port dredging waste in the production of coating mortars

**DOI:** 10.1007/s11356-026-37561-x

**Published:** 2026-03-07

**Authors:** Isabela Devesa Batista, Juliana Fadini Natalli, Markssuel Teixeira Marvila, Juliane Castro Carneiro, Luiz Gustavo Cruz Henriques da Silva, Afonso R. G. de Azevedo

**Affiliations:** 1 Advanced Materials Laboratory (LAMAV), State University of the Northern Rio de Janeiro (UENF), Campos Dos Goytacazes, RJ Brazil; 2 Universidade Federal de Viçosa (UFV), Campus Rio Paranaiba, Rio Paranaiba, MG Brazil; 3 Porto do Açu – Cais Açu Lab / Coletivo de Ações em Inovação e Sustentabilidade, Campos dos Goytacazes, RJ Brazil; 4 Civil Engineering Laboratory (LECIV), State University of the Northern Rio de Janeiro (UENF), Campos Dos Goytacazes, RJ Brazil

**Keywords:** Coating mortar, Port dredging waste, Ecological mortar

## Abstract

The accelerated expansion of the cement industry, driven by the growing housing demand in urban centers, has resulted in increased consumption of raw materials and elevated carbon dioxide (CO_2_) emissions. Simultaneously, the disposal of dredged material from ports and navigation channels presents an environmental concern, as improper management can significantly harm marine ecosystems. This study investigates the use of port dredging waste (PDW) as a supplementary cementitious material in coating mortars acting as a filler material, promoting packing and nucleation. Two types of cement were used: Ordinary Portland Cement (OPC) and Calcium Sulfoaluminate Cement (CSA), with PDW partially replacing cement at 10%, 30%, and 50% in mass, along with a control mix without replacement. The experimental program included evaluations of fresh and hardened properties, such as consistency (flow table and plunger tests), bulk density, entrained air content, water retention, flow, hydration kinetics by isothermal calorimetry, capillary water absorption, compressive and tensile strength, adhesion to substrates, and durability under wet-dry cycles. Characterization of the PDW indicated that it is appropriate for use as a filler material in coating mortars. In the fresh state, the incorporation of PDW reduced workability due to increased water demand, as observed in consistency tests. In the hardened state, replacing 10% of OPC with PDW improved compressive strength from 4.06 to 4.59 MPa and tensile strength from 0.3461 to 0.5796 MPa. In contrast, CSA-based mixtures showed reduced mechanical performance and durability as PDW content increased. These results suggest that 10% PDW replacement in OPC-based mortars is a viable and sustainable alternative, while further research is needed to address technical challenges in CSA-based systems. Practically, the use of PDW in OPC mortars offers an environmentally friendly strategy to reduce cement consumption, valorize dredged materials, and enhance the sustainability of construction coatings.

## Introduction

According to United Nations projections, by 2050, approximately 70% of the global population will reside in urban areas, a substantial increase that presents major challenges for urban infrastructure. This trend will necessitate the construction of a wide range of essential facilities, including residential buildings, schools, and hospitals, thereby intensifying the demand for natural resources and placing additional pressure on the construction industry. The sector’s expansion is directly linked to increased mortar usage, which drives higher consumption of cement and aggregates and, consequently, greater production and extraction of raw materials. This unbridled extraction process can lead to severe environmental impacts due to the progressive depletion of natural resources. In addition, the production of Portland cement generates significant carbon dioxide (CO_2_) emissions, worsening the global climate crisis (Nasr et al. 2020). In Brazil, the cement industry contributes approximately 2.6% of national CO_2_ emissions (da Silva Rego et al. 2023), reflecting the adoption of several mitigation strategies within the sector. Despite these efforts, cement production still generates around 572 kg CO_2_ per ton of cement (da Silva Rego et al. 2023), highlighting the need for further emission-reduction measures. This information makes it clear that supplementary cementitious materials are needed.

At the same time, dredging watercourses is an essential activity for maintaining adequate navigation depth (Chen et al. 2019), generatingPort dredging waste (PDW). However, the high volume of dredged material generated globally has become an environmental problem, especially when its disposal is carried out inappropriately, impacting aquatic ecosystems and water quality (Crocetti et al. 2022). In Brazil alone, studies highlight that approximately 80 million m^3^ of port dredging waste is generated annually, while other countries such as France and the UK generate 174 million and 103 million m^3^, respectively, demonstrating that this is a global concern (Bishnu Pada Bose and Moumita Dhar 2022). This material, when properly treated, has the potential to be used in mortars, as a substitute for Portland cement or other binders. In this context, some authors evaluated the possibility of using PDW as a substitute for mineral binders, as highlighted below: Dang et al. (2013) replaced cement with dredged marine sediments. Before the substitution, these materials were subjected to heat treatment at 650 °C and 850 °C. The results at a temperature of 650 °C showed better mechanical performance, in addition to being more advantageous in terms of energy savings. Laoufi et al. (2016) replaced cement with calcined dam mud, using temperatures of 750 °C, 850 °C, and 950 °C, and found better mechanical strength at a temperature of 750 °C. 

Aoual-Benslafa et al. (2015) found pollutants in its dredged sediment and had to treat it before use. After treatment and decontamination of the sediment, the 5% replacement was found to be the most viable. Rozière et al. (2015) also needed to decontaminate the sediments used in his research, and one of the methods used was calcination at 650°C. After treating the waste, the cement was replaced by 25%, achieving satisfactory results. Frar et al. (2017) used sediment dredged from two ports and replaced the cement by 20%, finding a significant loss of strength when compared to the reference. Finally, it suggests thermal activation of sediments to improve pozzolanic activities. Zhao et al. (2018) used dredged marine sediment as a substitute for cement; the treatment given to the waste was simply drying at 40 °C and grinding. There was a decrease in mechanical strength for higher replacement levels, which, according to the author, was due to the dilution of the cement when part of it was replaced by nonreactive or slightly reactive material. The dredged sediments must be analyzed for their composition and can normally be used in the construction industry in the production of concrete or mortars (Zhao et al. 2018).

Although previous studies have investigated the use of port dredging waste (PDW) in cementitious materials, they have primarily focused on its application as a partial replacement for Ordinary Portland Cement (OPC). In contrast, the present study evaluates the use of PDW as a partial substitute for both OPC and Calcium Sulfoaluminate Cement (CSA), representing a novel contribution. The replacement of CSA with PDW is primarily intended to promote a filler effect, enhancing particle packing and providing additional nucleation sites for hydration products. Furthermore, unlike earlier research that employed calcination treatments, this study utilizes PDW in its natural form, reducing processing requirements.

The relevance of this work lies in its contribution to the state of the art by addressing a type of waste that remains underexplored in the international literature, while also advancing sustainability in construction materials. Other research has analyzed untreated fine sediments as filler in concrete production (Beddaa et al. 2022). However, no studies were found with similar analyses in the production of rendering mortars, nor studies evaluating the combined use of CSA and PDW. This research aligns with the United Nations Sustainable Development Goals (SDGs), particularly SDG 11 (Sustainable Cities and Communities) and SDG 9 (Industry, Innovation, and Infrastructure).

## Materials and methods

The materials used in this study included Ordinary Portland Cement (OPC), composed of 6–10% limestone filler and 90–94% clinker and gypsum; Calcium Sulfoaluminate Cement (CSA), from the FRAGUAMAX brand, characterized by rapid setting and high early strength; type CH III hydrated lime; washed river quartz sand (maximum characteristic dimension = 1.18 mm), sourced from Campos dos Goytacazes, Rio de Janeiro, Brazil; and port dredging waste (PDW). OPC and lime were selected as conventional binders for coating mortars (Valdez Madrid et al. 2026), while CSA cement was included as a low-carbon alternative with distinct hydration kinetics, enabling the evaluation of PDW under different conditions.

PDW was obtained in sludge form, oven-dried at 105 °C for 24 h, ground in a ball mill, and sieved through a 0.150 mm mesh. This drying temperature was selected because it is sufficient to remove free water while minimizing changes to the material, thereby preserving the chemical characteristics of the PDW (Bhusal et al. 2025; Endale et al. 2025; Zhang et al. 2025). This particle size was selected based on literature reports indicating that particles finer than 0.150 mm enhance the filler effect by improving particle packing and matrix densification (Siddika et al. 2021). Notably, the material was used without calcination, in contrast to most previous studies. PDW was characterized by chemical composition, mineralogical composition, physical properties and morphology.

The chemical composition of PDW, OPC and CSA cements was obtained using the X-ray fluorescence (XRF) test. The test was carried out using a Shimadzu EDX-700 equipment. The mineral composition was obtained by X-ray diffraction (XRD) using a monochromatic Cu-Kα radiation detector and nickel at a speed of 1.5°/min, 2θ scanning ranging from 5 to 60°. The particle size distribution was obtained by laser granulometry using a Microtrac s3500 equipment under wet conditions and morphology analysis was carried out using scanning electron microscopy (SEM) by Jeol microscope model JSM 6460 LV.

Tests were carried out with the mortar in the fresh state, in order to study its workability, consistency, and the handling quality of the mortar produced. Eight types of coating mortar were produced in a 1:1:6 ratio (cement:lime:sand) in mass. Two types of cement were used in the mortars, in addition to different percentages of cement replacement by waste, according to Table 1.

**Table 1:** Compositions used with Ordinary Portland Cement and Calcium Sulfoaluminate Cement

Composition	Port dredging waste (g)	Ordinaty Portland Cement (g)	Calcium Sulfoaluminate Cement (g)	Lime (g)	Sand (g)	Water (g)	Water/cement
OPC0	-	100	-	100	600	145	1.45
OPC10	10	90	-			155	1.55
OPC30	30	70	-			165	1.65
OPC50	50	50	-			180	1.80
CSA0	0	-	100			155	1.55
CSA10	10	-	90			155	1.55
CSA30	30	-	70			165	1.65
CSA50	50	-	50			185	1.85

In the fresh state, the following analyses were performed: consistency index, NBR 13276 (ABNT 2016); density in the fresh state, NBR 13278 (ABNT 2005a). incorporated air, NBR 13278 (ABNT 2005a); water retention, NBR 13277 (ABNT 2005b); squeeze flow, NBR 15839 (ABNT 2010); and calorimetry, ASTM C1679 (ASTM International 2014). All tests were performed in triplicate, using three experimental samples.

The proportions studied were subjected to tests in the hardened state, in order to understand the mechanical and absorption properties of the mixtures. The test specimens were produced and molded according to NBR 13279 (ABNT 2005c) and demolded after 48 h. After demolding, the samples were maintained at room temperature for 28 days, and at this age they were subjected to the proposed tests. The waste was blended with the other constituents to ensure mixture homogeneity.

In the hardened state, the following analyses were performed considering the application of coating mortars: mass density in the hardened state, NBR 13280 (ABNT 2005d); water absorption by capillarity, NBR 15259 (ABNT 2005e); water absorption by immersion, NBR 9778 (ABNT 2009); compressive strength and tensile strength in flexure, to NBR 13279 (ABNT 2005c); tensile adhesion strength, NBR 13528 (ABNT 2019). All tests were performed in triplicate, using three experimental samples.

The durability test using wetting and drying cycles is essential to evaluate the performance of the mortar in adverse weather conditions, considering the continuous exposure to precipitation and evaporation processes throughout its useful life (Dehestani et al. 2020). This test’s main objective is to simulate the aging of the material and test its strength after being subjected to adverse conditions.

Each cycle lasted 24 h: (i) 1 h in an oven at 60 °C, considering a high-temperature situation affecting the mortar; (ii) 0.5 h after the oven, at room temperature of 25 °C, to regulate the temperature of the specimen; (iii) 22 h submerged in water; and (iv) 0.5 h after submersion, at room temperature of 25 °C to regulate humidity. This test was carried out after 28 days of curing of the specimens in 0, 30, and 60 cycles. At the end of the cycles, the specimens were subjected to compressive strength and tensile flexural strength tests, following the other research (Baptista Junior et al. 2024). In addition to the wetting and drying cycles in distilled water, the test was carried out in saline solution (Table 2), with the same process of carrying out the test and analyzing the same parameters.

**Table 2:** Composition of saline solution for durability tests

Chemical compound (salt)	Quantity (g/L)
Sodium Chloride P.A.—NaCl	30.0
Magnesium Chloride P.A.—MgCl2.6H2O	6.0
Magnesium Sulfate P.A.—MgSO4	5.0
Calcium Sulfate P.A.—CaSO4	1.5
Hydrogenated Potassium Carbonate P.A.—KHCO3	0.2

After carrying out the tests in the fresh and hardened state as well as the durability tests, the best results were analyzed regarding their structure. This was done through the X-ray diffraction (XRD) test using the PROTO AXRD Benchtop scanning between 10° and 60° with a step of 0.015. The source (Cu-Kα tube) reaches a voltage of 30 kV and 20.5 mA.

## Results and discussions

### PDW characterization for application in coating mortar

Table 3 presents the chemical composition of PDW, OPC and CSA cements obtained by X-ray fluorescence (XRF) test. The PDW used in this research has, in higher percentages, silica (SiO_2_), alumina (Al_2_O_3_) and lime (CaO), as found by Frar et al. (2017). Results are consistent with research by Aoual-Benslafa et al. (2015) and Laoufi et al. (2016) who found silica and lime in greater quantities in the material. The chemical composition of the material reveals a combined content of SiO_2_, Al_2_O_3_, and Fe_2_O_3_ greater than 70%, suggesting its potential to present pozzolanic activity (Mota et al. 2016). However, pozzolanic activity needs to be activated, which normally happens at high temperatures, through calcination of the material. This did not occur in the present study, since PDW was used as a filler material. Its behavior is therefore comparable to that of limestone filler, with the primary objective of enhancing sustainability by reducing greenhouse gas emissions and overall energy consumption.

**Table 3:** X-ray fluorescence of materials used in this research

Material	SiO2	Al2O3	CaO	Fe2O3	Cl	SO3	K2O	TiO2
Port dredging waste	34.26	28.84	15.00	11.99	3.88	2.18	1.89	1.46
Ordinary Portland Cement	17.79	4.36	66.50	2.76	0.25	4.63	0.48	0.27
Calcium Sulfoaluminate Cement	9.09	14.11	63.17	0.82	-	11.24	0.71	0.71

**Fig. 1:**
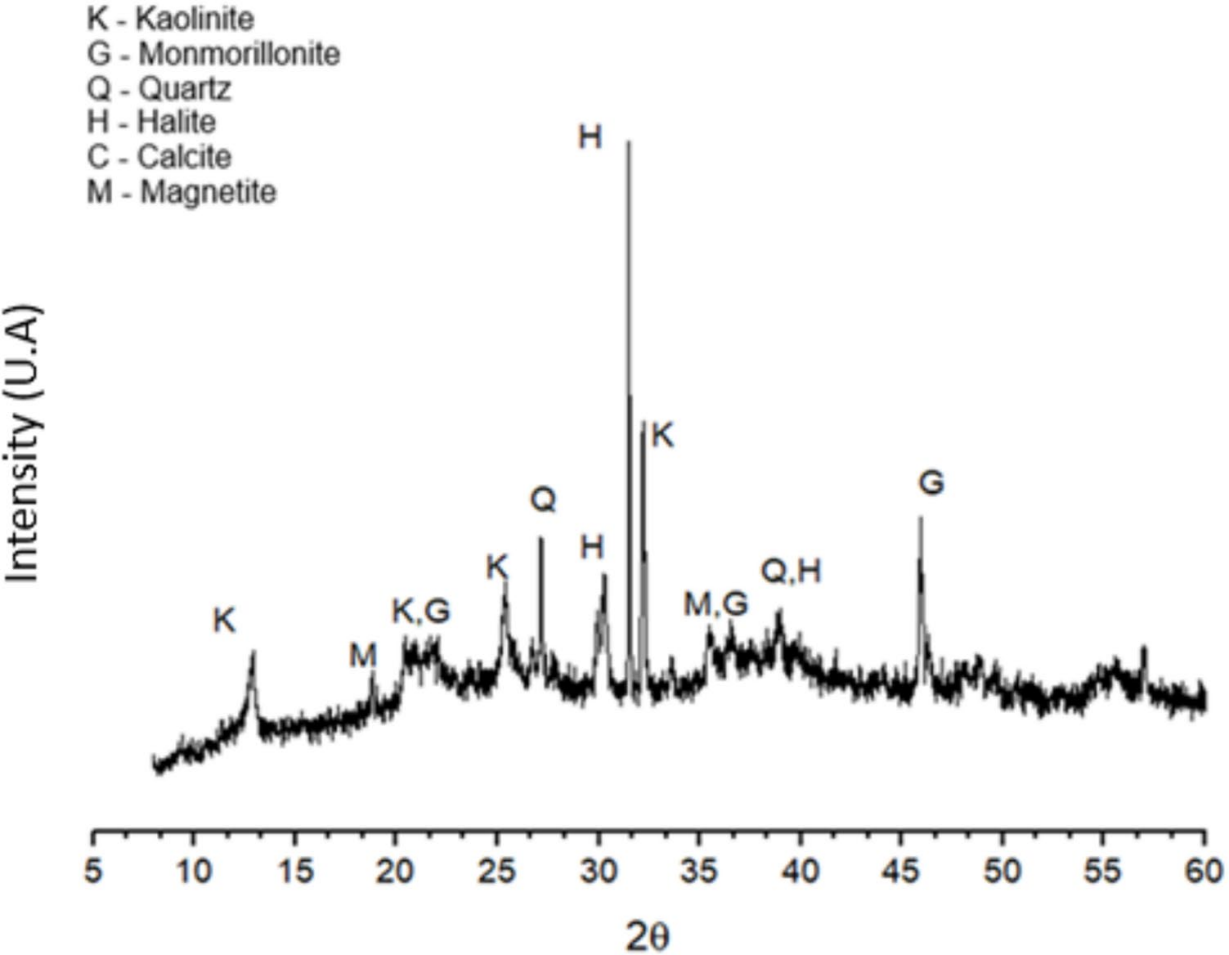
X-ray diffraction of the port dredging waste

The XRD (Fig. 1) demonstrates that the PDW has mineralogical phases of kaolinite, montmorillonite, quartz, halite, and magnetite. The absence of mineral and organic pollutants in the PDW is evidenced in both Fig. 1 and Table 3. This finding is consistent with the literature, which reports that such materials are predominantly composed of mineral fractions (sand, silt, clay, and carbonates), with relatively low contents of organic matter and organic contaminants (Carreira et al. 2025; Ferrans et al. 2019). The peaks between 30° and 33° are halite (NaCl), a phase that is not present in the composition of cements. The presence of NaCl is due to the origin of the dredged material. Although the dredging takes place in the internal channel’s oath port, these channels are connected to the sea, and the vessels themselves can bring salt in their hulls. According to EN 206–1 (EN 206–1, 2000), the chloride content in cement-based materials must not exceed 0.4% of the cement mass, which does not occur in any of the replacement percentages studied, with the 50% replacement coming closest to the maximum allowed limit. Another important point is that OPC is capable of fixing lower chloride levels due to the presence of aluminous phases, such as C_3_A and C_4_AF. These components are capable of promoting the formation of Friedel’s Salt, which, in addition to fixing the chloride level, reducing durability problems, may be capable of improving the mechanical strength of the hardened material (Shi et al. 2017; Wang et al. 2025). The same occurs for CSA, a sulfo-aluminous cement that also promotes the fixation of free chlorides (Jiang et al. 2024). Therefore, it is worth noting that, since the chloride levels present in PDW are low, the material can be used as a replacement for OPC and CSA without problems related to the chloride level. However, due to the essentially inert nature of PDW, its application as a binder substitute is inherently limited. While its incorporation is beneficial in terms of nucleation effects and improved particle packing, PDW should be used in controlled and relatively low replacement levels when substituting cementitious binders.

**Fig. 2:**
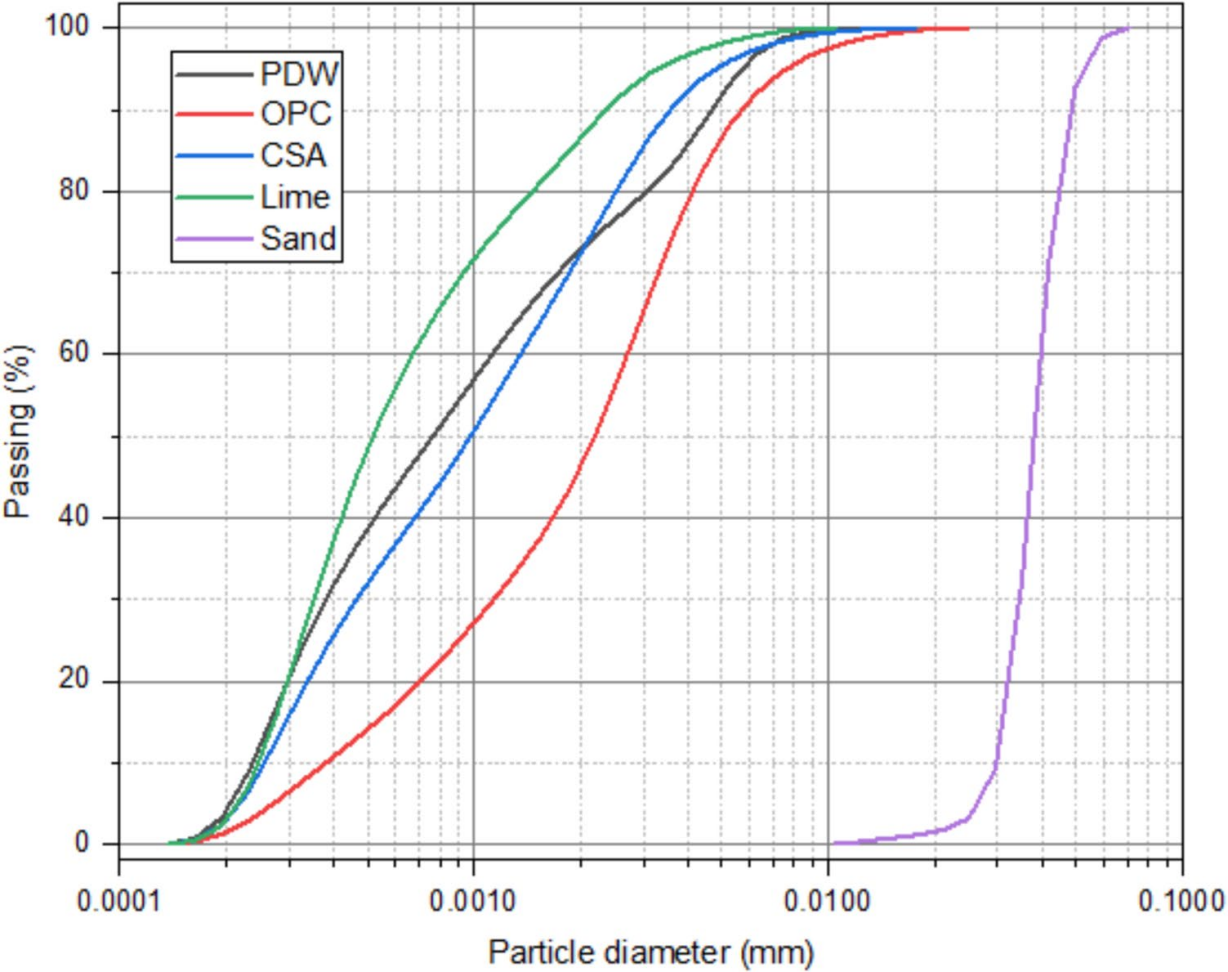
Granulometry of the materials used for the production of mortars

Figure 2 presents the particle size distribution of the materials used in this study. The results show that PDW particles are predominantly finer than those of the cement. The analysis was performed using a Microtrac S3500 particle size analyzer, operating with dry dispersion. Based on the particle size distribution, PDW can be classified as silty clay. According to EN 12620 (British Standards Institution, 2008), it qualifies as a filler material, as over 70% of its particles are smaller than 0.063 mm, thus allowing it to perform a filler function in cementitious mixtures, as highlighted in the article’s objectives.

**Fig. 3:**
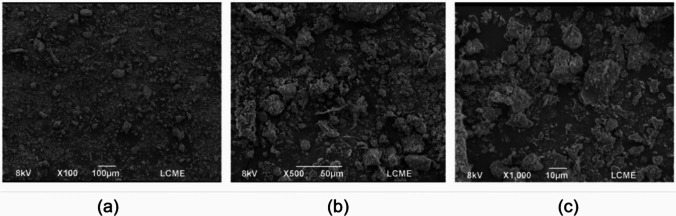
Scanning electron microscopy of the port dredging waste: **a** 100× zoom; **b** 500× zoom; **c** 1000× zoom

Figure 3 presents the scanning electron microscopy (SEM) analysis of the PDW, revealing angular particles of varying sizes with rough surfaces. The increased specific surface area of the material contributes to higher water demand to achieve adequate workability in mortar mixtures (Rozière et al. 2015). The particle size distribution depends on the treatment the waste underwent (Rozière et al. 2015), in this case, the waste was prepared and passed through a sieve with an opening of 0.075 mm; therefore, the largest particle is a maximum of 74 mm.

### Fresh state tests

The consistency index test made it possible to determine the water/cement ratio of each of the proportions studied, and it is possible to observe, in Table 1, that the need for a greater quantity of water increased as the replacement content increased. This trend may occur due to the finer particle size of the material when compared to cement (Zhao et al. 2018). Ideally, the amount of kneading water should be as small as possible, as excess water leads to greater porosity and lower mechanical strength (Silva et al. 2010). Still according to Zhao et al. (Zhao et al. 2018), the fact that the waste retains more water means that the amount of free water is smaller, resulting in a significant loss in the workability of the mortar. Therefore, it is necessary to increase the amount of water in the mixture to achieve the same workability as the reference mortar, as can be seen in Table 1. However, the fineness of PDW can be optimized or combined with chemical admixtures to mitigate the additional water demand. Strategies include optimizing the particle size distribution by limiting excessive ultrafine fractions (< 75 µm), blending different PDW size ranges to improve particle packing and reduce void content, and using polycarboxylate-based superplasticizers (PCEs) to enhance particle dispersion and maintain workability at lower water contents (Duan et al. 2025; Z. Wang et al. 2024a, b).

**Fig. 4:**
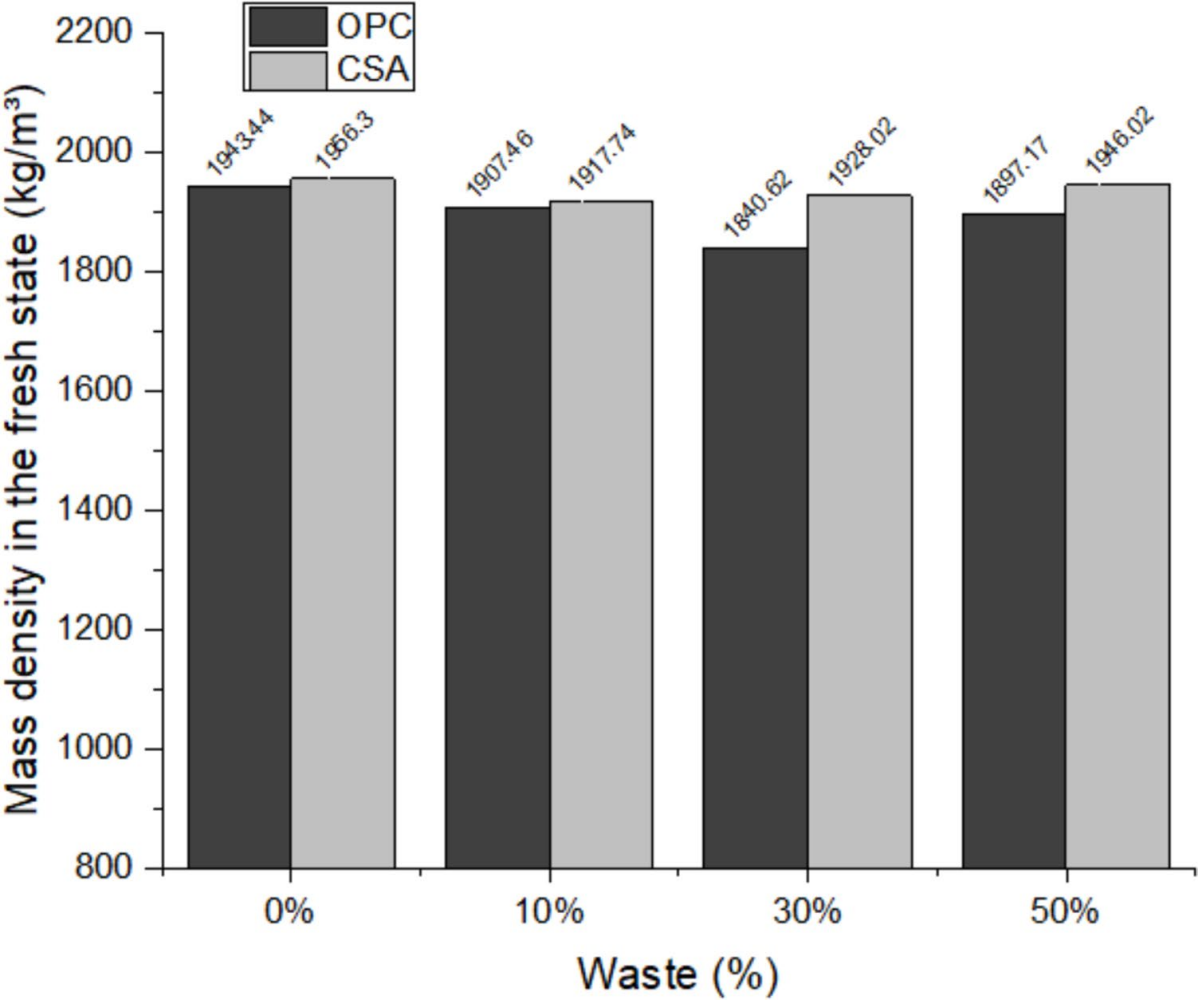
Fresh mass density results of the mortars

Figure 4 presents the results of fresh mass density. The density of the mixtures using OPC cement decreased slightly as the replacement content increased, up to a percentage of 30%, corroborating the results found by Zhao et al. (2018). In general, there is no great variation in density, due to the similarity between the density of the materials, the dredging waste has a specific density of around 2.8 g/cm^3^ (Rozière et al. 2015), while OPC cement has 2.96 g/cm^3^ and CSA 2.93 g/cm^3^.

**Fig. 5:**
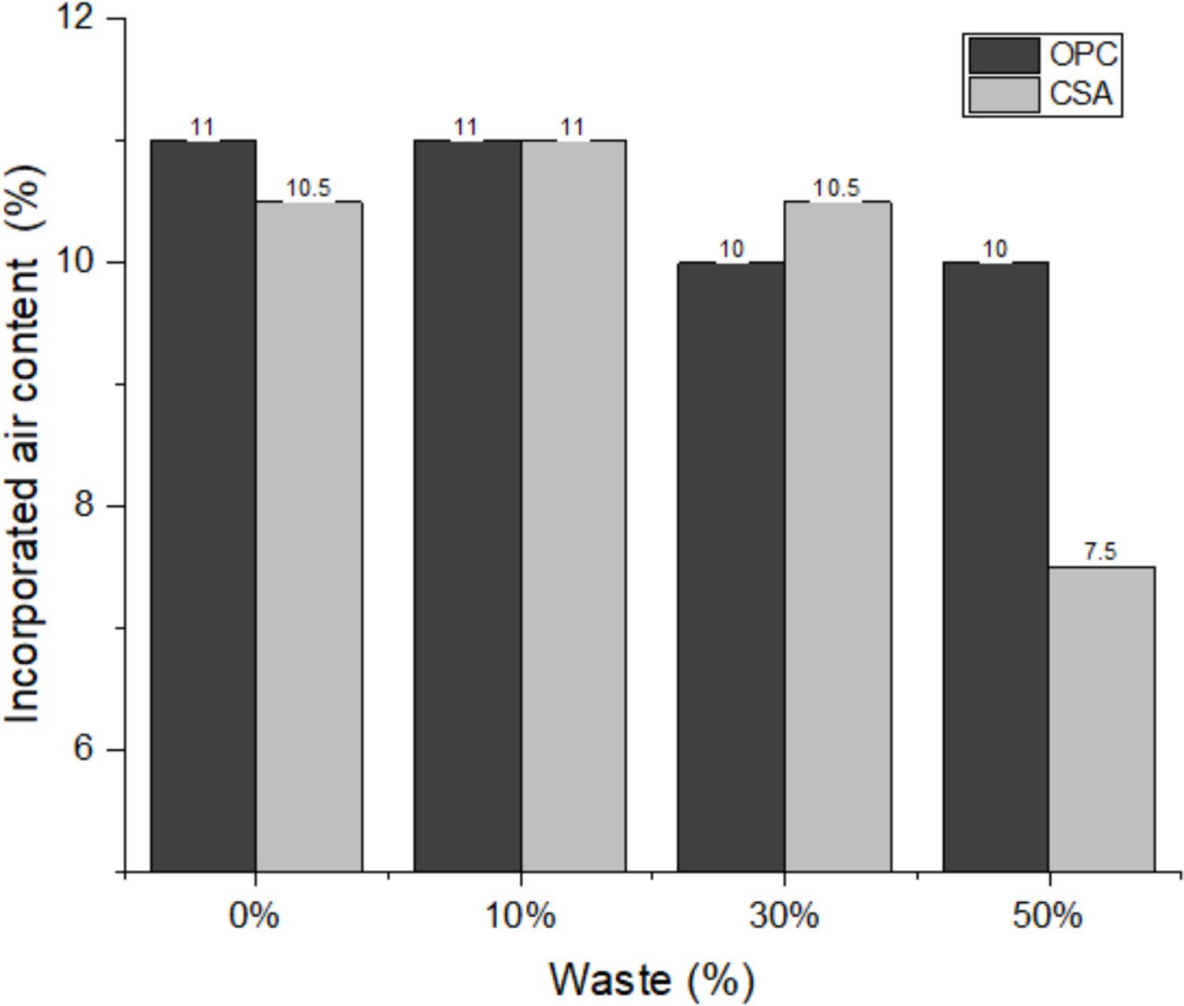
Incorporated air content of the mortars

The incorporated air content of mortars is shown in Fig. 5. This is a complex property because it is related to the density of the material in the fresh state and water retention (Tolmachov et al. 2019). In general, there are no specific prescribed limits for incorporated air content according to particular exposure conditions, such as humid or frost-prone environments. However, it is well established that excessively high air contents lead to reductions in mechanical strength, whereas excessively low air contents may impair the mortar’s flexibility and ability to accommodate deformations (Choi et al. 2016). For this reason, several authors recommend an incorporated air content in the range of 8–17% for coating mortars, as this interval represents a compromise between strength and deformability (Santos et al. 2018). It can be observed that all compositions present values of incorporated air within this limit, with the exception of the 50% CSA composition. This may occur for two reasons: (i) first, this is the composition with the highest amount of water, as shown in Table 1; with the increase in the amount of water, less space is available for the incorporated air; and (ii) the CSA forms resistant phases faster than the OPC, rapidly forming ettringite (Chang et al. 2024; S. Wang et al. 2024a, b). With the influence of this phase, which is voluminous and expansive, and the effect of PDW, used at 50% in this composition, there was a reduction in the incorporated air from 11% (0% CSA composition) to 7.5%.

When comparing mixtures that have the same type of cement, the incorporated air content decreases with the increase in the replacement of cement with waste. This fact may be related to the filler effect of the material, in which the empty spaces are filled, reducing the air content in the mortar (Durante Ingunza et al. 2018). The decrease in air content with increasing replacement is very beneficial for the mortar, as it indicates that mortars may have lower open porosity (Kameche et al. 2023), indicating that these should have less water absorption due to immersion and greater durability. This decrease in air content can be attributed to the filling of empty spaces with fine material, which tends to reduce the amount of trapped air, a result that corroborates what was found by other authors (Parghi and Shahria Alam 2016) (Jubeh et al. 2019).

**Fig. 6:**
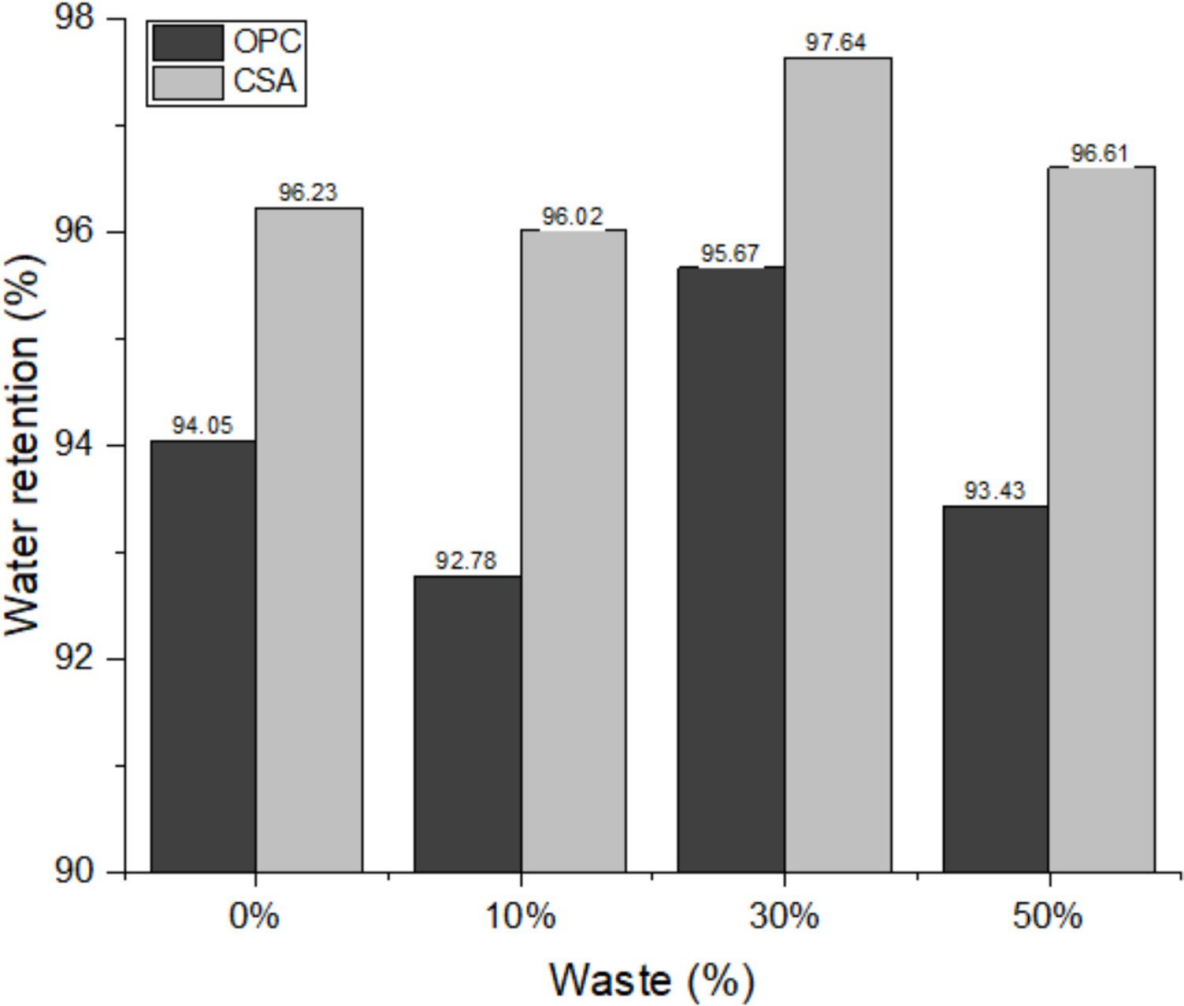
Water retention of the mortars

Figure 6 shows the water retention results of the mortars. Water retention in mortar is the material’s ability to retain mixing water against suction from the base or against evaporation (Leone et al. 1998). This mixing water is responsible for making the mortar hardening reactions more gradual, causing hydration to occur properly, which interferes with the material’s gain in mechanical strength and influences adhesion to the substrate. Water retention can influence the shrinkage or expansion of the mortar after its drying, as greater retention reduces water evaporation, and consequently its loss to the substrate (Durante Ingunza et al. 2018).

Retention, in the same replacement percentage, increases in the different cases studied, always being lower in compositions containing OPC cement and higher in CSA. This is because the CSA reaction is faster and forms ettringite, a bulky phase that retains water (Chang et al. 2024; S. Wang et al. 2024a, b). However, when comparing the different replacement percentages of the same cement, the values fluctuate, not showing linear behavior (Fig. 6). This nonlinearity can be attributed to the combined effects of particle packing, filler action, and changes in pore size distribution caused by the progressive incorporation of the waste. At lower replacement levels, the filler effect may be insufficient to significantly modify the pore structure, while at higher contents, excessive dilution of the cementitious phase may limit hydration and reduce water-binding capacity (Huang et al. 2021; Kaptan et al. 2024). The maximum water retention occurs at 30% PDW replacement for both cements, indicating an optimal balance between filler-induced densification, ettringite formation, and water availability, which enhances cement hydration compared to other replacement levels.

**Fig. 7:**
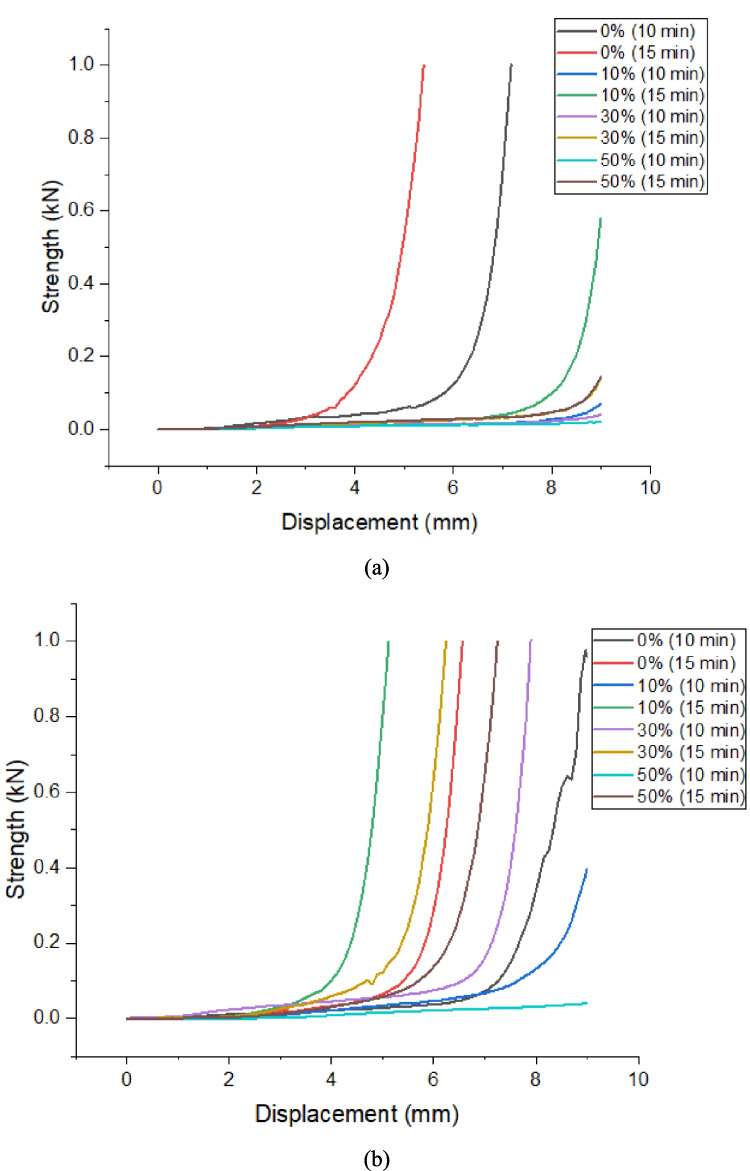
Squeeze flow results in compositions using **a** Ordinary Portland Cement and **b** Calcium Sulfoaluminate Cement

Figure 7a, b shows the results of the rheological analysis using the squeeze flow test, under different test execution conditions. The behavior of compression flow can be divided into three stages: the first being characterized by linear elastic behavior where there is small tension applied; the second in which plastic deformation occurs, which is when the material deforms considerably with a modest increase in force applied; and the third which is known as strain hardening, where the load increases rapidly (Cardoso et al. 2009)(Min et al. 1994). Through this test it is possible to visualize the fluidity behavior of the mixture under axial load conditions, as occurs in the laying of mortar. Initially, it must be indicated that if the displacement is greater for higher waste replacement levels, under the same load conditions and speed rate, it means that the waste offers greater plasticity to the mixture.

The fluidity of the mortar in the squeeze flow test is linked to the air content incorporated in the mixture; therefore, the smaller the amount of voids, the greater the plasticity. This can be seen in Fig. 7, where some of the mixtures using OPC cement reach the maximum displacement of the test without reaching the third stage. This facilitates the application and finishing of the material. Mixtures using CSA cement require a greater load for a smaller displacement; this fact may be related to the setting time of this cement, which begins approximately 15 min after hydration, due to the formation of ettringite (Deng et al. 2022; Liu et al. 2024). Compositions using CSA cement may be limited due to the rapid hydration that this system presents, with a lot of water loss making fluidity difficult (Costa et al. 2020). In compositions using OPC cement, there may be an improvement in the packaging of the material, making the mixture behave according to the nature of the waste, creating a filling effect. In general, the fluidity of the mixtures was changed with the presence of the waste, increasing its viscosity depending on the type of cement used and the content of waste replaced.

**Fig. 8:**
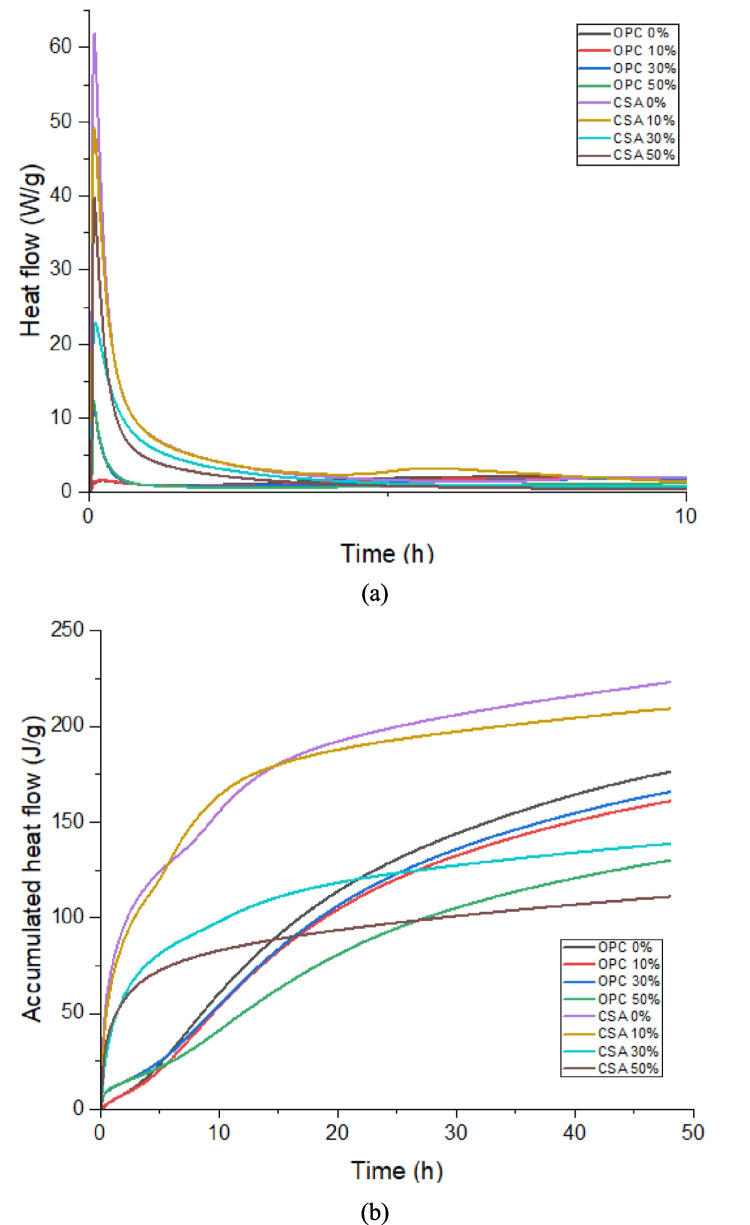
**a** Heat flow results found by calorimetry test; **b** results of total accumulated heat found by the calorimetry test

Figure 8a shows the heat flow monitored up to 10 h. Mixtures with the same base composition demonstrated consistent results with increasing substitution content. Mixtures using OPC cement have the lowest heat flux values, presenting values between 1 and 13 W/g, where the percentage of 10% presents a lower result than the reference and higher heat flux in percentages of 30 and 50%, respectively, inversely to what occurs in CSA cement mixtures, where the heat flow decreases with the increase in the replacement of cement with waste. The values found in the mixtures using CSA cement showed peaks between 23 and 62 W/g, with curves that demonstrate accelerated hydration. In other words, the incorporation of PDW generally increases the heat flow in OPC-based compositions by providing additional nucleation sites, which accelerate hydration (Jansen et al. 2012; Schöler et al. 2017). Conversely, in CSA-based compositions, the addition of PDW reduces heat flow because CSA hydration is already rapid and dominated by ettringite formation; in this case, PDW acts as a diluent, decreasing the proportion of reactive cement and consequently lowering the overall heat release as the replacement level increases (Zhang et al. 2021).

In general, the curve shifts to the left with an increase in the replacement content, which indicates that replacement can accelerate the cement hydration process (Hamdadou et al. 2023), in addition to achieving lower heat flow in most compositions. Although it is difficult to quantitatively determine the exact acceleration time induced by the incorporation of PDW in OPC and CSA-based cementitious materials, this effect is clearly observed in Fig. 8. This acceleration can be attributed to several mechanisms. Previous studies indicate that the filling material can act as a nucleation site for cement hydration products (Deboucha et al. 2021)(Deboucha et al. 2022). Heterogeneous nucleation in cement hydration refers to the process in which the initial formation of hydration products occurs preferentially around fine particles or impurities, accelerating the hydration process (Isaia and Rizzatti, 2021). Causing an increase in reactions and changes in the type of hydration products formed, as will be highlighted by the XRD results discussed later in the text. In this way, a greater number of crystals are formed instead of a reduced number of larger crystals (Mehdipour et al. 2017), since there is less cement due to replacement.

It is possible to observe that the total heat released, Fig. 8b, decreases with increasing replacement, a result that is in accordance with the literature, since when filling is added to the mortar, the cement dilution effect occurs (Mounanga et al. 2011) (Deboucha et al. 2021). The mixtures using OPC cement present a curve similar to that found by Frolich et al. (Frølich et al. 2016). Specifically dealing with compositions with CSA cement, the use of PDW is an efficient strategy to dissipate the high heat of hydration typical of this type of cement, where the heat reduces from approximately 230 J/g (CSA composition 0%) to around 100 J/g (CSA composition 50%).

### Hardened state tests

**Fig. 9:**
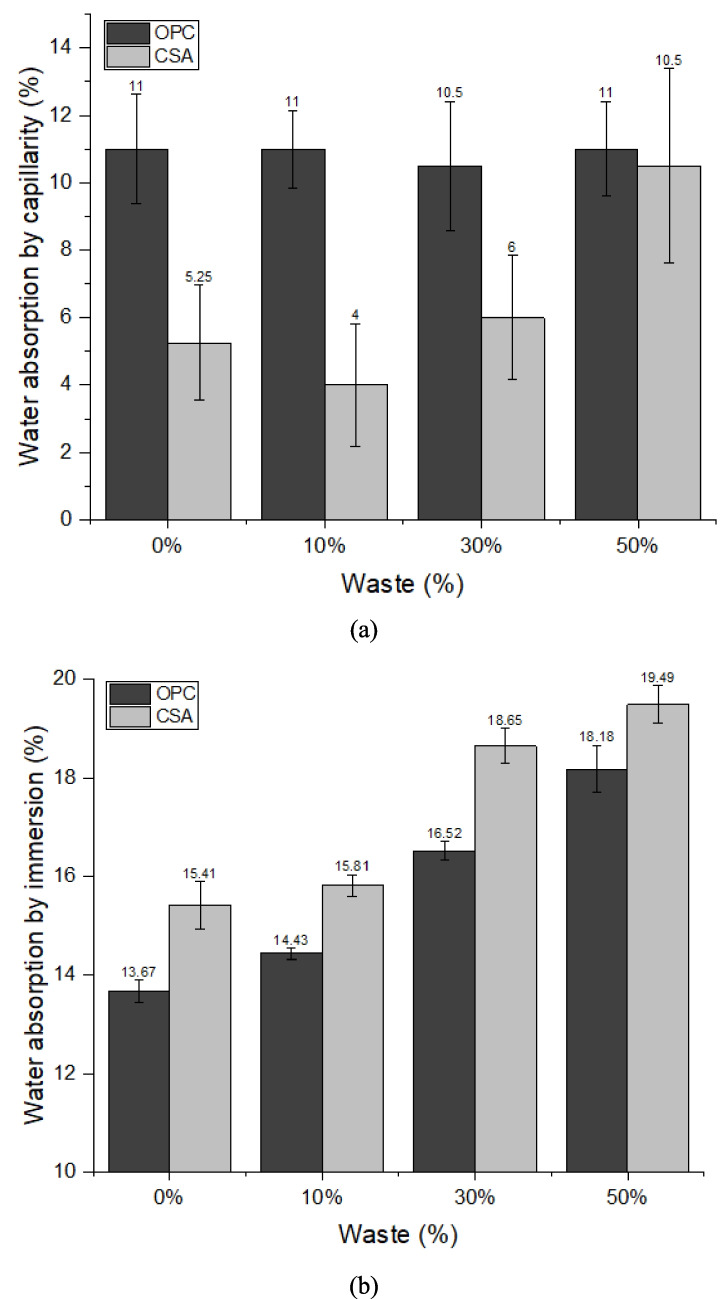
Water absorption results of the mortars by: **a** capillarity and **b** immersion

Figure 9a presents the results of capillarity water absorption. In the absorption of water by capillarity, the vertical flow of water occurs, which ascends through the pores present (Yedra et al. 2022). These pores are interconnected, forming capillaries. The rise occurs at different heights, depending on water evaporation and the porosity of the material (Lailson 2023) and other factors such as pore size and pore size distribution (Marvila et al. 2022), which makes this property intrinsically complex. Capillary absorption tends to be greater when there is a higher w/c ratio (Lozano-Lunar et al. 2019), corroborating the fact that a higher w/c ratio indicates a decrease in mechanical strength, as well as greater absorption by capillarity also indicates lower mechanical strength. This fact suggests that a structure with more connected pores has less strength (Lozano-Lunar et al. 2019). It can be observed that, for OPC-based compositions, the addition of PDW did not significantly affect water absorption by capillarity, as the results were comparable within the error bars shown in Fig. 9a. This behavior would likely differ if PDW exhibited pozzolanic activity rather than acting solely as a filler. Analysis of the chemical composition of PDW (Table 3) shows that it is rich in SiO_2_, Al_2_O_3_, and CaO, which could potentially react in cementitious systems. However, these components are present in crystalline forms (kaolinite, quartz, calcite, and montmorillonite, as shown in Fig. 1) rather than in amorphous, reactive forms. This explains why the incorporation of PDW did not lead to a reduction in capillary water absorption in OPC-based mortars.

In Fig. 9a, it can be seen that the mixtures containing CSA cement have lower absorption, showing that they are denser and less porous materials, which can contribute to greater mechanical strength, as can be seen in the air content, density and flexural tensile strength and compressive strength. Meanwhile, this same mixture of CSA cement presents a similar amount of pores to the other compositions when subjected to immersion absorption, demonstrating that it has a regular porosity, between 8 and 17%, but these are not interconnected. The fact that capillarity remains or decreases with increasing incorporation content demonstrates that the waste can perfectly supply the dilution of the cement in this test, indicating that the waste can fill the voids and interrupt the capillaries, being a satisfactory result for the application proposed in this research.

Figure 9b shows the results of water absorption by immersion. Water absorption by immersion is directly linked to the number of voids present in the mortar and the ability of these pores to absorb water when submerged (Lozano-Lunar et al. 2019). The waste has a smaller particle size; therefore, it has a larger specific surface area when compared to the cements studied, which results in finer pores and greater absorption (Hendrickx et al. 2010). In Fig. 9b, it is possible to observe that mortars manufactured using a higher replacement percentage absorb more water and are consequently more porous (Lozano-Lunar et al. 2019). In this test, the specimens are subjected to extreme conditions, where they are completely submerged until their pores are filled with water, demonstrating that the compositions using OPC cement have fewer pores and that there is no significant difference between the reference and 10% replacement. Less water absorption in the mortar gives it greater durability, as aggressive agents from the environment need to penetrate the mortar to damage it. However, compositions containing CSA cement present greater water absorption through immersion, both in the 0% and 50% composition, which is attributed to the greater porosity of the mortars containing this binder. It should be noted that the lack of more detailed information on the porosity characteristics of mortars containing PDW (size, quantity, and distribution), as well as of the different compositions produced with OPC and CSA cements, limits a deeper discussion of the observed differences in behavior. This aspect therefore represents a limitation of the present study.

**Fig. 10:**
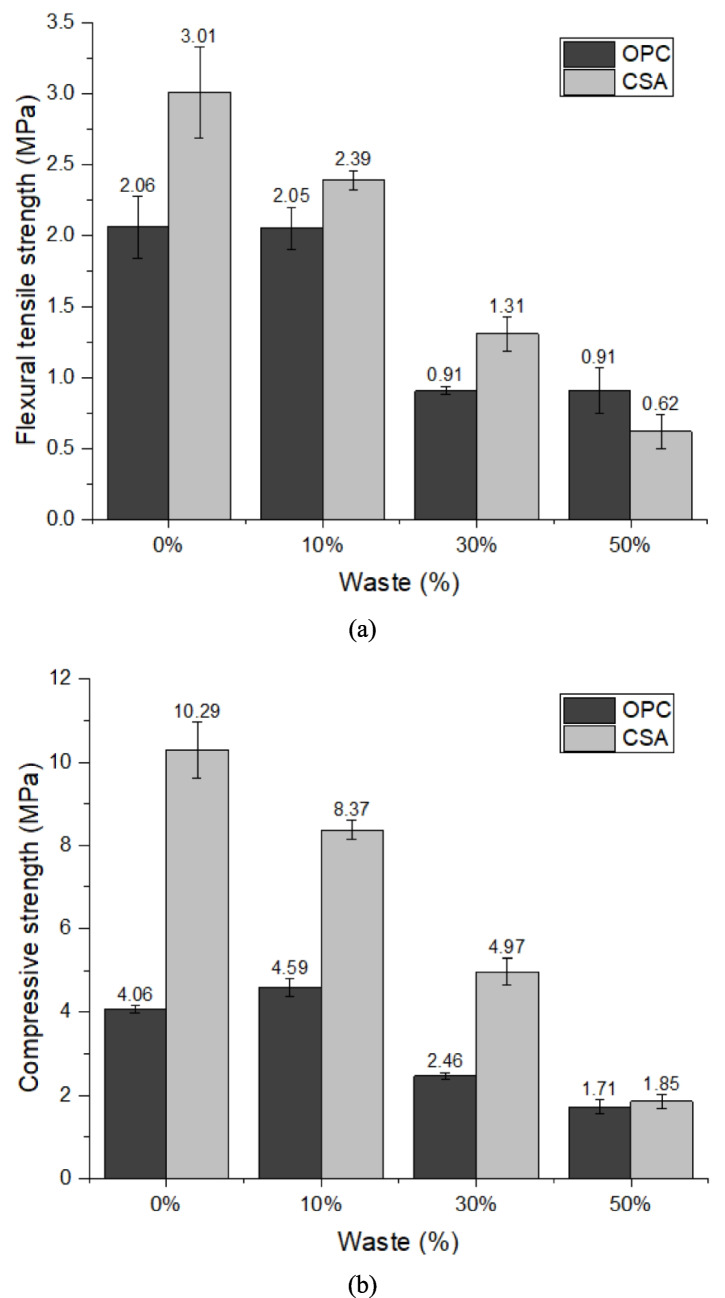
Mechanical strength results of the mortars at 28 days: **a** flexural tensile strength and **b** compressive strength

Figure 10a, b presents the results found in the flexural tensile strength and compressive strength, respectively. Flexural tensile strength is extremely important for the coating mortars studies in this research, being necessary that it reaches at least 1 MPa of strength, which happens for all cases when replacing 0 and 10%, in addition to meeting the requirements in 30% replacement using CSA cement. Compressive strength must be at least 1.5 MPa, a value achieved in all compositions studied. The drop in strength with increasing replacement content demonstrates that the cement was replaced by nonreactive or slightly reactive material (Zhao et al. 2018), indicating that the material offers filling properties (Rozière et al. 2015) and not cementitious or pozzolanic properties as found by other authors (Dang et al. 2013) (Laoufi et al. 2016). Another important point is that there was an increase in the amount of water/cement ratio as the amount of PDW increased (Table 1). It is known that the water/cement ratio is inversely proportional to compressive strength (Marvila et al. 2020), which is why there may have been a reduction in this property. Therefore, the use of superplasticizers could be a viable strategy to maintain the same water/cement ratio across all compositions, potentially enhancing the mechanical strength of higher-water-content mixtures, such as OPC30 and OPC50. The strength found, for the most part, met the requirements, demonstrating that the waste filled the pores and supplied, to a certain extent, the reduction in the amount of cement. The use of PDW is interesting as a supplementary cementitious material.

Another point worth highlighting is that CSA-based coating mortars exhibit higher mechanical strength than OPC, even with PDW replacement. This is due to CSA promoting rapid ettringite formation, lower sensitivity to dilution, and the filler effect of PDW, which enhances particle packing (Chen et al. 2025; Park et al. 2024; Zhang et al. 2021). These factors explain the superior compressive strength values observed for CSA0 (10.29 MPa), CSA10 (8.37 MPa), and CSA30 (4.97 MPa), as shown in Fig. 10b. It is also noteworthy that the OPC10 composition (4.59 MPa) exhibits higher strength than the reference OPC0 (4.06 MPa), a trend not observed in CSA-based compositions. This improvement is attributed to the nucleation effect, which accelerates the initial dissolution of OPC and enhances compressive strength. As shown in Fig. 8a, OPC10 also exhibits increased heat flow compared to OPC0, confirming that nucleation promotes both faster hydration and higher strength in the composition containing 10% PDW as a partial OPC replacement.

**Fig. 11:**
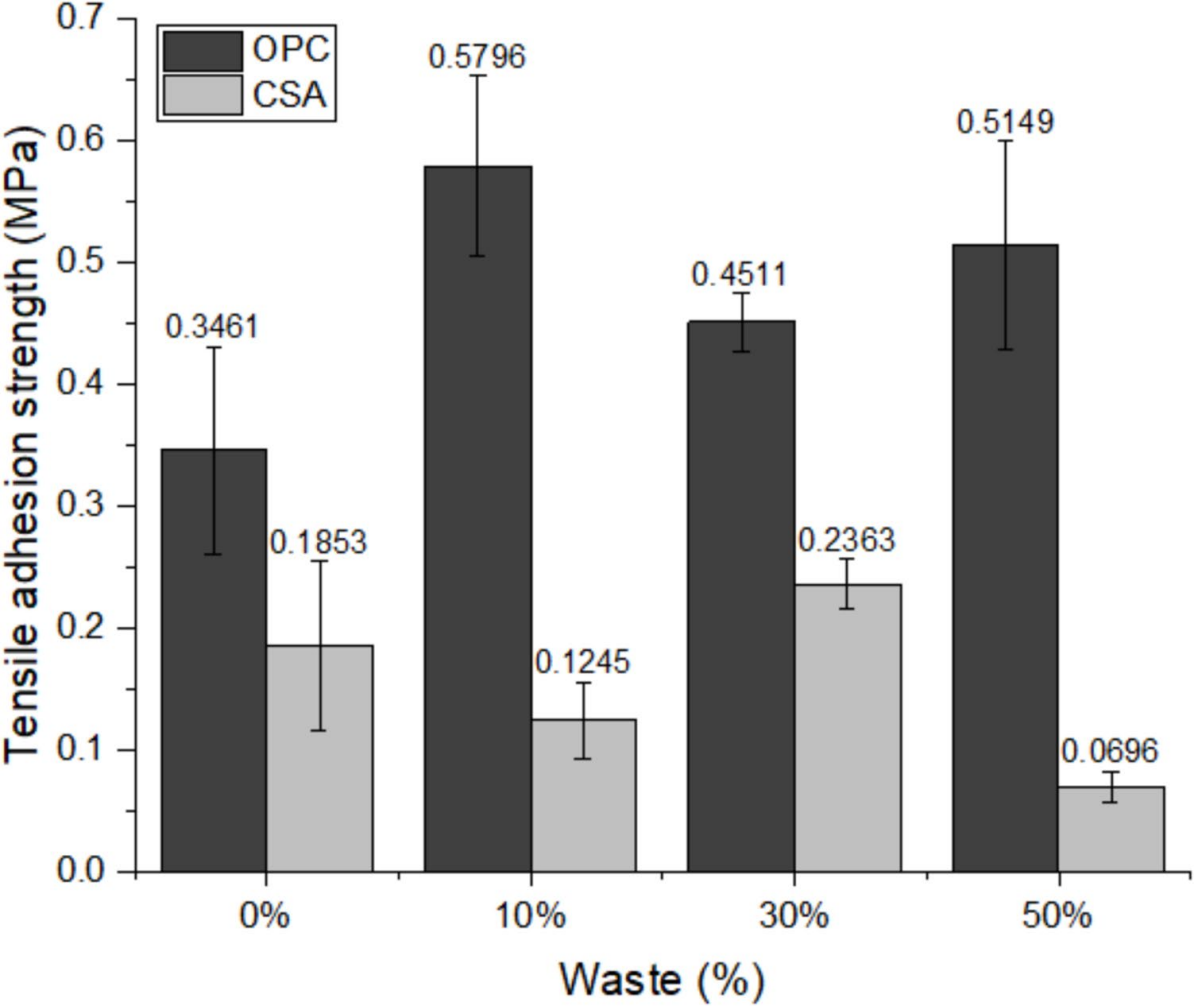
Tensile adhesion strength results of the mortars at 28 days

The tensile adhesion strength of the mortar is the property of the coating to resist the stresses acting at the interface with the substrate (Vaz and Carasek 2019). The minimum value allowed for external application or receipt of ceramic coating is 0.3 MPa by NBR 13279 (ABNT 2013). Therefore, all compositions using OPC cement meet the requirement, while none of the compositions using CSA do, and only the 30% composition can be used on the ceiling, which allows a minimum strength of 0.2 MPa (Fig. 11). The results corroborate water retention, which demonstrates that values between 75 and 95% present the best results, having better adhesion to the substrate, and consequently better strength.

### Durability test

After the cycles, the materials were subjected to flexural tensile strength and compressive strength tests, requiring them to meet the minimum values permitted by standard, which are 1 MPa and 1.5 MPa, respectively. In general, the mixtures showed better results after 30 durability cycles, which may be related to the curing time of the mortars, indicating that at 28 days of age, when the test began, the chemical reactions had not yet been completed (Lozano-Lunar et al. 2019); thus, the increase in curing time may have refined the structure of the pores present in the material (Cui and Cahyadi 2001), thus increasing the mechanical strength.

**Fig. 12:**
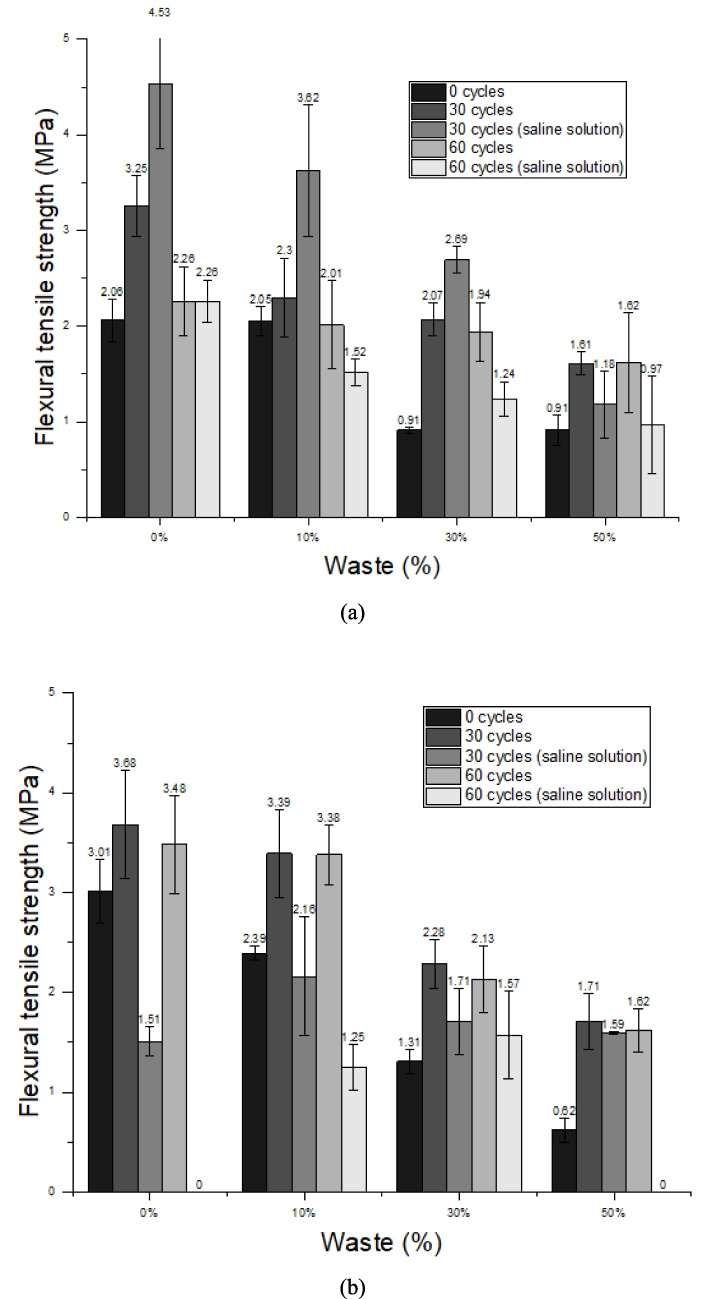
Flexural tensile strength results of the mortars after durability test: **a** Ordinary Portland Cement and **b** Calcium Sulfoaluminate Cement

The mixtures using OPC cement showed the best results in flexural tensile strength when compared to mixtures with CSA cement. Using OPC cement, only mixtures of 0 cycles with 30 and 50% replacement and 60 cycles in saline solution with 50% replacement do not meet the requirements, as can be seen in Fig. 12a.

Figure 12b shows the results obtained in flexural tensile strength using CSA cement, indicating that this composition does not present good results when subjected to contact with saline water for a longer period of time, resulting in no sample available to carry out the test, a fact that occurred at percentages of 0 and 50%, after 60 cycles. During the hydration of CSA cement, substantial quantities of ettringite are produced. This ettringite undergoes a chemical transformation through its interaction with chloride ions (Cl^−^), leading to the formation of 3CaO·Al_2_O_3_·CaCl_2_·10H_2_O. This reaction effectively binds free chloride ions, thereby enhancing the chloride-binding capacity of the matrix and contributing to improved durability against chloride-induced degradation (Huang et al. 2024). However, when PDW contains chlorides, the amount exceeds the binding capacity of CSA, promoting severe degradation. This effect is reinforced by internal expansion resulting from the formation and destabilization of ettringite and monosulfate phases, as well as by phase dissolution, resulting in a significant reduction in mechanical strength. Pretreatment of PDW, such as washing to remove soluble chlorides, could improve compatibility with CSA, reducing chloride-induced degradation.

**Fig. 13:**
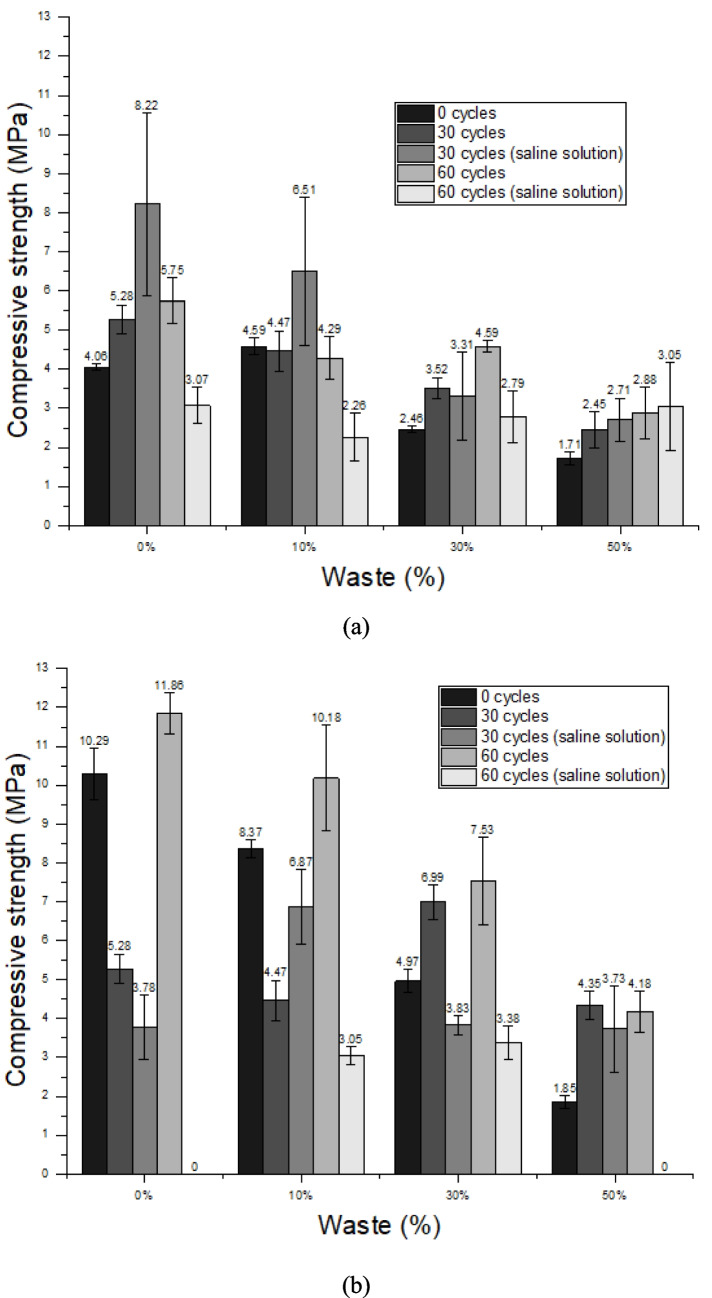
Compressive strength results of the mortars after durability test: **a** Ordinary Portland Cement and **b** Calcium Sulfoaluminate Cement

Unlike the flexural tensile strength, in the compressive strength the best results found were not in the mixtures using OPC cement; however, it was still the one that presented constant results, with no loss of any composition before the end of the test. All results were satisfactory, as they reached the minimum value of 1.5 MPa required by the standard, as shown in Fig. 13a. The results found in the compressive strength of mixtures using CSA cement were the best; however, this composition does not behave in a beneficial way when exposed to saline water. This fact occurs in mixtures with 0 and 50% replacement. The compositions that reached the end of the test met the requirements, Fig. 13b. In general, the increase in the replacement content reduced the mechanical strength after the durability cycles, similar to what occurred in the mechanical strength tests carried out when the mortar was 28 days. Indicating that, in terms of mechanical strength, the PDW was not able to compensate for the lack of cement so effectively. However, some of the compositions still met the requirements and can be used.

**Fig. 14:**
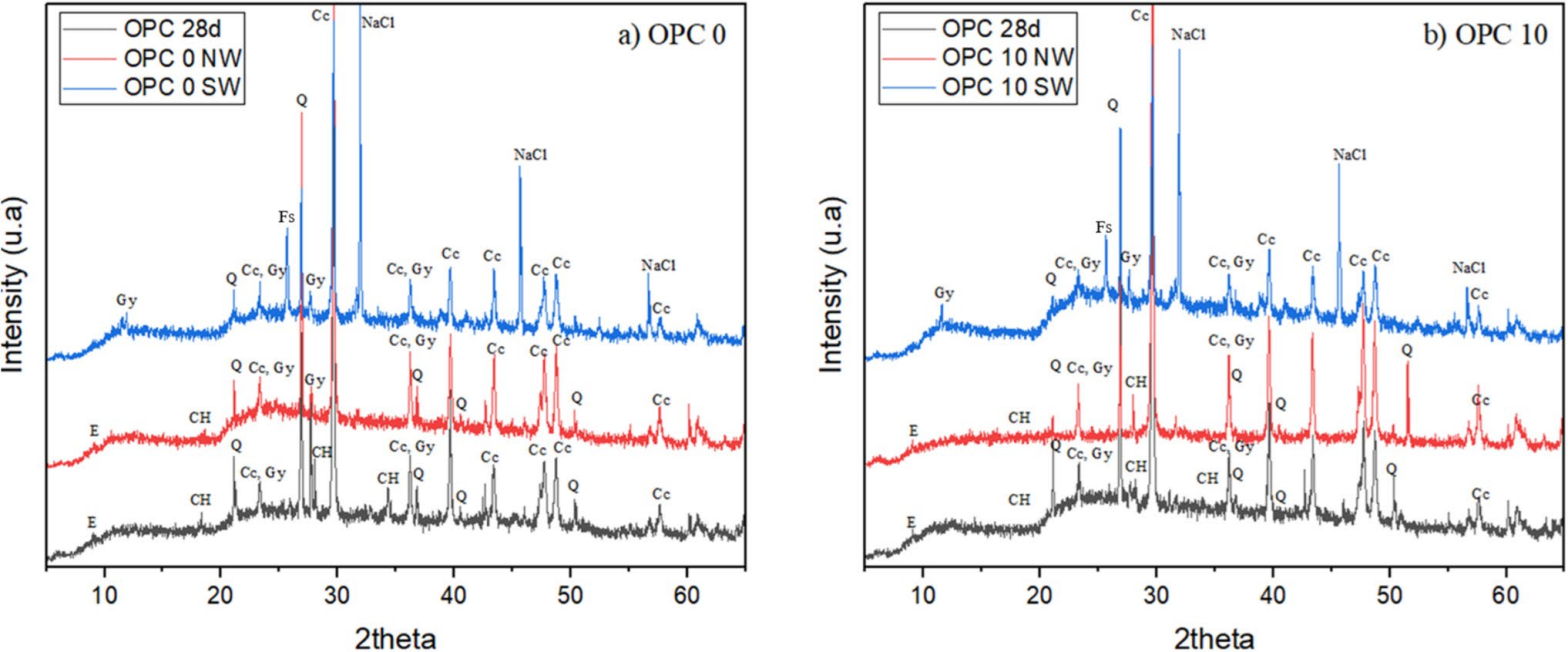
X-ray diffraction (XRD) patterns of mortar samples at 28 days and after 60 durability cycles: **a** 0% port dredging waste; **b** 10% port dredging waste. Legend: Q = quartz (SiO_2_); CH = portlandite [Ca(OH) _2_]; E = ettringite [Ca₆Al_2_(SO₄) _3_(OH) ₁_2_·26H_2_O]; Fs = Friedel’s salt [Ca_2_Al(OH) ₆Cl·2H_2_O]; Cc = calcium carbonate (CaCO_3_); Gy = gypsum (CaSO₄·2H_2_O); NaCl = free chloride (NaCl)

The analysis of the material’s microstructure was carried out only on the best composition found after carrying out the other tests and on the reference sample since more compositions were studied and some of them did not meet the minimum requirements. XRD was carried out on samples made of OPC cement with 0 and 10% replacement at 28 days of age and after passing through 60 durability cycles in normal water (NW) and saline water (SW), as highlighted in Fig. 14.

It can be observed that the 0% compositions without durability exposure (28 days) and those exposed to normal water (NW) exhibit the presence of ettringite (E) and portlandite (CH), which are not detected in the 0% composition exposed to saline water (SW). A similar behavior is observed for the 10% composition, suggesting that ettringite and portlandite were partially consumed due to chloride interaction during the durability exposure. This behavior helps to explain the mechanical results presented in Fig. 13a, in which compressive strength values of 4.06, 5.28, and 8.22 MPa are observed for the 0% compositions without exposure (28 days) and after 30 cycles in normal water and saline water, respectively.

One possible explanation for this behavior is chloride binding promoted by reactions with ettringite and portlandite, leading to the formation of Friedel’s salt (da Silva Junior et al. 2026; Homayoonmehr et al. 2022). Nevertheless, free chloride (NaCl) is still detected in the 0% and 10% compositions exposed to saline water (SW), indicating that chloride binding was not complete. This result justifies the reduction in mechanical strength observed after 60 durability cycles, as shown in Fig. 13a.

Other crystalline phases identified in Fig. 14 include quartz (Q), associated with the use of sand in the mixtures, as well as gypsum (Gy) and calcium carbonate (Cc), related to the use of OPC in the compositions. These phases are more pronounced in the diffractograms, particularly for the 0% composition, indicating a higher cement content and, consequently, greater reactivity and mechanical strength for this mixture.

### *Quantification of CO*_*2*_* eq/ton of cement*

To perform the analysis of CO_2_ eq/ton of cement, the following baseline values were adopted from the literature: 783 kg CO_2_ eq/ton for OPC (He et al. 2019); 508.95 kg CO_2_ eq/ton for CSA, estimated indirectly by assuming that CSA presents approximately 35% lower CO_2_ emissions than OPC under conservative conditions (Tanguler-Bayramtan et al. 2024); and 50 kg CO_2_ eq/ton for PDW, based on published reports for dredged port materials similar to those investigated in this study (Martine Kox et al. 2021). It should be emphasized that this analysis considers only the binder fraction involved in mortar production, accounting for the mass replacement of OPC or CSA by PDW, while excluding emissions associated with sand, lime, water, transportation, or any other logistical or processing stages. Based on these assumptions, the results presented in Table 4 were obtained.

**Table 4:** CO_2_ eq/ton of cement for compositions of the mortars

Composition	CO_2_ eq/ton of cement	Relative %
OPC0	783.00	100.00%
OPC10	709.70	90.64%
OPC30	563.10	71.92%
OPC50	416.50	53.19%
CSA0	508.95	65.00%
CSA10	463.06	59.14%
CSA30	371.27	47.42%
CSA50	279.48	35.69%

A reduction in CO_2_ eq per ton of cement of 90.64% was observed for the OPC10 composition, 71.62% for OPC30, and 53.19% for OPC50. When compared to the reference OPC0 composition, the CSA50 mixture exhibited the most favorable performance from the perspective of CO_2_ eq per ton of cement, corresponding to only 35.69% of the emissions associated with OPC0. These results clearly highlight the importance of incorporating supplementary cementitious materials, such as PDW, as partial replacements for industrial binders in order to significantly reduce the carbon footprint of cement-based materials.

## Conclusion

This study assessed the potential of using port dredging waste (PDW) as a partial replacement for cement in rendering mortars through a comprehensive experimental program. The results showed that PDW has a high water absorption capacity, leading to increased water demand in mixtures with higher replacement levels and a reduction in entrained air content, especially when combined with CSA cement, suggesting interactions between PDW and binder type. From a mechanical standpoint, only mixtures with 10% cement replacement met the minimum requirements for both flexural and compressive strength according to regulatory standards and literature references, although the mixture with 30% CSA cement also achieved satisfactory strength levels. Regarding tensile adhesion, all mixtures with OPC cement performed adequately for external applications and for substrates receiving additional coatings. In contrast, the mixture with CSA cement and 30% replacement was only suitable for internal ceiling applications due to lower adhesion values. These findings support the technical feasibility of incorporating PDW in mortars, particularly at a 10% replacement level with OPC cement, which was the only condition to meet all assessed performance criteria.

As a suggestion for future work, it is recommended: (i) to perform durability tests using other methods, such as sulfate attack and carbonation, in mortars produced with OPC and CSA containing PDW; (ii) to evaluate other PDW replacement contents, such as 5%, 20%, and 40%, and analyze the effects on the properties of the coating mortars; (iii) to evaluate other relevant parameters for mortars with PDW, like shrinkage deformation or modulus of elasticity; (iv) to evaluate parameters of porosity (size, distribution, and quantity) of mortars containing PDW produced with OPC and CSA; and (v) to evaluate the optimization of PDW particle size for application in mortars produced with OPC and CSA.

## Data Availability

No data was associated with this study.
